# The cascade of care for latent tuberculosis infection in congregate settings: A national cohort analysis, Korea, 2017–2018

**DOI:** 10.3389/fmed.2022.927579

**Published:** 2022-09-15

**Authors:** Jinsoo Min, Hyung Woo Kim, Helen R. Stagg, Molebogeng X. Rangaka, Marc Lipman, Ibrahim Abubakar, Yunhee Lee, Jun-Pyo Myong, Hyunsuk Jeong, Sanghyuk Bae, Ah Young Shin, Ji Young Kang, Sung-Soon Lee, Jae Seuk Park, Hyeon Woo Yim, Ju Sang Kim

**Affiliations:** ^1^Division of Pulmonary and Critical Care Medicine, Department of Internal Medicine, Seoul St. Mary’s Hospital, College of Medicine, The Catholic University of Korea, Seoul, South Korea; ^2^Division of Pulmonary and Critical Care Medicine, Department of Internal Medicine, Incheon St. Mary’s Hospital, College of Medicine, The Catholic University of Korea, Seoul, South Korea; ^3^Usher Institute, The University of Edinburgh, Edinburgh, United Kingdom; ^4^Institute for Global Health, University College London, London, United Kingdom; ^5^UCL-TB, University College London, London, United Kingdom; ^6^UCL Respiratory, Division of Medicine, University College London, London, United Kingdom; ^7^Royal Free London NHS Foundation Trust, London, United Kingdom; ^8^Department of Urology, Seoul St. Mary’s Hospital, College of Medicine, The Catholic University of Korea, Seoul, South Korea; ^9^Department of Occupational and Environmental Medicine, Seoul St. Mary’s Hospital, College of Medicine, The Catholic University, Seoul, South Korea; ^10^Department of Preventive Medicine, College of Medicine, The Catholic University of Korea, Seoul, South Korea; ^11^Department of Internal Medicine, Cheju Halla General Hospital, Jeju-si, Jeju-do, South Korea; ^12^Division of Pulmonary and Critical Care Medicine, Department of Internal Medicine, Ilsan Paik Hospital, Inje University College of Medicine, Goyang-si, South Korea; ^13^Division of Pulmonary Medicine, Department of Internal Medicine, Dankook University College of Medicine, Cheonan-si, South Korea

**Keywords:** preventive therapy, quality control, social worker, school teacher, student, latent TB infection (LTBI)

## Abstract

**Background:**

In 2017, Korea implemented a nationwide project to screen and treat latent tuberculosis infection (LTBI) in high-risk for transmission public congregate settings. We aimed to assess programme success using a cascade of care framework.

**Materials and methods:**

We undertook a cohort study of people from three congregate settings screened between March 2017 and December 2018: (1) first-grade high school students, (2) employees of educational institutions, (3) employees of social welfare facilities. We report percentages of participants with LTBI completing each step in the cascade of care model. Poisson regression models were used to determine factors associated with not visiting clinics, not initiating treatment, and not completing treatment.

**Results:**

Among the 96,439 participants who had a positive interferon-gamma release assay result, the percentage visiting clinics for further assessment, to initiate treatment, and who then completed treatment were 50.7, 34.7, and 28.9%, respectively. Compared to those aged 20–34 years, individuals aged < 20 years and aged ≥ 65 years were less likely to visit clinics, though more likely to complete treatment once initiated. Using public health centres rather than private hospitals was associated with people “not initiating treatment” (adjusted risk ratio [aRR], 3.72; 95% confidence interval [CI], 3.95–3.86). Nine-month isoniazid monotherapy therapy was associated with “not completing treatment,” compared to 3-month isoniazid and rifampin therapy (aRR, 1.28; 95% CI, 1.16–1.41).

**Conclusion:**

Among participants with LTBI from three congregate settings, less than one third completed treatment. Age, treatment centre, and initial regimen were important determinants of losses to care through the cascade.

## Background

Latent tuberculosis infection (LTBI) is defined as a state of persistent immune response to stimulation by *Mycobacterium tuberculosis* antigens with no evidence of clinically manifest active TB. LTBI is a large reservoir for active TB because 5–10% of those infected will develop active TB over their lifetime. Approximately one-quarter of the world’s population is estimated to have LTBI (1). Its treatment is a critical component of the World Health Organization’s (WHO) End Tuberculosis (TB) Strategy (2). Our ambitious target of TB elimination by 2050 is only achievable if we prevent new TB infection and reduce pre-existing pool of LTBI, in addition to control active TB cases (3). As such, the WHO has highlighted the importance of expanding the screening and treatment of LTBI, especially in low-incidence countries (4, 5). However, its strategies to tackle LTBI have been underscored worldwide. The coronavirus disease 2019 pandemic has imposed unprecedented impact to the healthcare system including TB preventive measures (6).

Tuberculosis remains a serious public health problem in the Republic of Korea. In 2020, Korea has its incidence rate of 49 per 100,000 population with the highest TB burden among the high-income countries (7, 8). From 2011 to 2016, with the strengthening of TB prevention and care policies (9) and the implementation of a public-private collaboration model (10), a 5.2% annual national reduction in the incidence of newly-reported TB was achieved. However, TB outbreaks continued to occur in various congregate settings, such as schools, neonatal intensive care units, postpartum care centres, and social welfare facilities, resulting in a significant societal burden of disease (11, 12). The Korean government designated these facilities as high-risk for TB congregate setting as their densely populated and confined environments could drive TB infection (13). In 2016, the Korean TB Prevention Act was revised to include mandatory TB and LTBI screening for employees in these congregate settings. In 2017, systematic LTBI testing were provided to approximately 1.2 million individuals (14).

As with all public health interventions, the introduction of new LTBI management as a public health intervention in Korea requires programme monitoring to ensure its quality, effectiveness, and impact (15). This is particularly important given that, globally, targetted approaches for LTBI testing among high-risk groups have often been recommended (4) rather than screening an unselected population, even within congregate settings.

The tuberculin skin test (TST), which has been used for years for the diagnosis of LTBI, has several limitations, such as poor specificity in persons vaccinated with Bacille Calmette–Guérin (BCG) and immunocompromised patients and cross-reactivity with environmental non-tuberculosis mycobacteria (16). Interferon-gamma release assay (IGRA) is a whole blood assay to detect the interferon-gamma produced *in vivo* by sensitised T cells after *in vitro* stimulation with mycobacterial antigens, which are not found in BCG and most non-tuberculous mycobacteria, and thus its specificity for *M. tuberculosis* is higher than with the TST. Because of high BCG vaccination rate in Korea and concerns of false-positive reactions to the TST, the nationwide LTBI project utilised the IGRA alone strategy for LTBI diagnosis.

Cascade of care models are useful when evaluating of patient retention during the multiple steps of diagnostic and treatment pathways (17). Such cascades aid the quantification of gaps in care delivery and highlight areas that require quality-of-care improvements. In this manuscript, we perform the first full evaluation of the LTBI cascade of care for individuals screened in publically-utilised congregate settings, focussing on outcomes for people who tested positive by IGRA.

## Materials and methods

### Study design and data source

We constructed a prospective observational cohort of individuals screened in congregate settings within the nationwide screening project for LTBI (18). Individuals were tested by IGRA between March 2017 and December 2018. In order to create a “*TB FREE COREA (latent TuBerculosis inFection scREEning and treatment in COngREgAte settings)*” database of relevant data, we used and cross-linked four databases: the LTBI screening database from the government program, the national health information database, the public healthcare information system database, and the Korean national TB surveillance system (19). Anonymised joint keys, which are replacements for personal identification numbers, were used to link the LTBI screening database with the other three databases through deterministic matching.

### Study setting and participants

**FIGURE 1:**
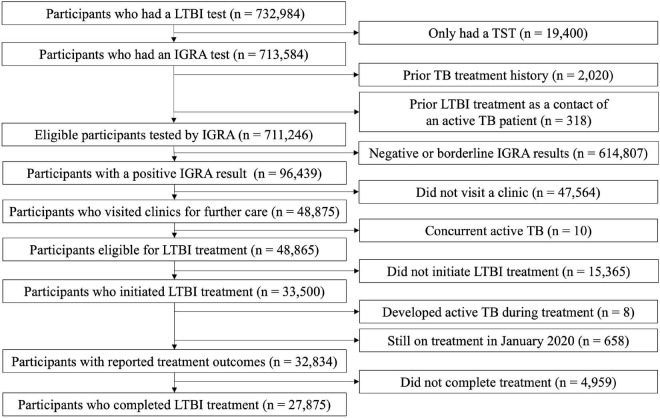
Flow chart of enrolled participants with positive results from an interferon-gamma release assay. LTBI, latent tuberculosis infection; TST, tuberculin skin test; IGRA, interferon-gamma release assay; TB, tuberculosis.

In this analysis, we included individuals screened within high-risk for TB congregate settings: (1) employees of educational institutions, such as child day care centres, kindergartens, primary schools, middle schools, and high schools, (2) employees of social welfare facilities, and (3) first-grade high school students (15–16 years old). The eligibility criteria included: (1) having undergone IGRA testing and (2) an absence of a prior TB treatment history. Those who received LTBI treatment previously because of being a close contact of an active TB patient were excluded. Among 732,984 people who had a LTBI test, we excluded 19,400 participants who only had undergone TST (Figure 1). Of 711,246 participants were screened by IGRA, 96,439 (13.6%) participants tested positive by IGRA and formed the final cohort of interest for this study.

### Systematic tuberculosis and latent tuberculosis infection screening process

Because of concerns of false positive reactions to the TST in Korea with high BCG vaccination coverage, the IGRA-only strategy was chosen as the first diagnostic method during the nationwide LTBI project. IGRA was performed using QuantiFERON-TB Gold In-Tube tests (Qiagen, Hilden, Germany) and the results were interpreted according to the manufacturer’s instructions. Those with positive IGRA results could freely choose to use either public health centres or private hospitals for further LTBI management (20, 21). LTBI screening and treatment were provided free of charge by the government. On visiting a treatment centre, patients were clinically assessed and underwent chest radiography according to national TB guidelines (22). After the exclusion of active TB disease, LTBI treatment was offered.

### Outcome variables

The specific outcomes of interest were: (1) the number of participants with positive IGRA results who visited clinics for further care, (2) the number of people who were given a prescription for LTBI treatment and started that treatment, and (3) the number of people who completed treatment. Three types of LTBI treatment regimens were recommend based on Korean guidelines (22): isoniazid (INH) monotherapy for 9 months (9INH), rifampin (RIF), monotherapy for 4 months (4RIF), and INH and RIF combination therapy for 3 months (3HR). The Korean government offers free LTBI treatment, and it is mandatory for physicians to enter the R76.80, which is a “Latent TB” based on the seventh edition of the Korean Classification of Disease (KCD-7), when offering free LTBI treatment under the current national health insurance system. This R76.80 is specifically used when physicians prescribe anti-TB drugs (INH, RIF, or both) for LTBI treatment. Thus, identifying the R76.8 code and prescribing anti-TB drugs can accurately guide to define participants under the LTBI treatment. Individuals were considered to have completed their treatment course if they were prescribed more than 80% of the total expected dose within 12 months for 9INH, 6 months for 4RIF or 4 months for 3HR (23, 24). Individuals were designated as “still on treatment” if their calculated treatment completion date was on or after January 1, 2020.

### Independent variables

We collected data on factors that may influence the cascade of care in LTBI treatment and diagnosis, such as sex, age, income level, place of residence, comorbidities, treatment centre, and initial treatment regimen. Income level was divided into 20th percentiles based on the amount of the national health insurance premium paid, ranging from the first (the lowest 5%) to 20th (the highest 5%) ventiles (25). This was then categorised into four groups: low (1st to 5th ventiles), lower-middle (6th to 10th), upper-middle (11th to 15th) and high (16th to 20th). Medical Aid beneficiaries (26) who do not pay a premium, were added to the lowest income tier. Place of residence was categorised into: rural area, small to medium-sized city, and metropolitan city. The Charlson Comorbidity Index, assigned based on the severity of each disease, was used to measure the extent of comorbidities (27). It comprises of a wide range of chronic conditions, such as coronary artery disease, congestive heart failure, chronic pulmonary disease, peptic ulcer disease, peripheral vascular disease, liver disease, cerebrovascular disease, connective tissue disease, diabetes, dementia, renal disease, leukaemia, lymphoma, solid tumour, and acquired immune deficiency syndrome. The KCD-7 and the 10th revision of the International Classification of Disease and Related Health Problems were the source of diagnostic codes. The treatment centre that individuals with positive IGRA results initially visited for LTBI management was categorised as either a public health centre or a private hospital. The implications of each independent variable and its data source has been described in the Supplementary Table.

### Statistical analyses

Discrete variables are presented as frequencies and percentages. The time taken for each step in the cascade of care were calculated and are presented as a median with an interquartile range (IQR) or a mean with a standard deviation (SD).

Next, we determined the relationship between our exposures of interest and three specific *a priori* determined outcomes of interest, i.e., “not visiting clinics,” “not initiating treatment,” and “not completing treatment.” We used univariable and then multivariable Poisson regression with a robust variance estimator to estimate the relative risk (SAS proc genmod/R package geepack). All exposures of interest were included in each multivariable model. We also conducted the multiple correspondence analysis to visualise the association between not completing LTBI treatment and explanatory variables. Statistical analyses were conducted with R v.3.5.2 (R foundation for Statistical Computing, Vienna, Austria) and SAS statistical software (version 9.4; SAS Institute, Cary, NC, USA).

### Extended analysis

In the model analysing factors associated with “not initiating treatment,” we conducted a subgroup analysis to assess the association between age and “not initiating treatment,” stratified by types of treatment centre. This was because quality of healthcare services between the public and private sectors are different in Korea (21, 28). We chose participants aged 20–34 years as the reference age group, because this age group is the most important target for preventive therapy. In the model analysing the factors associated with “not completing treatment,” we conducted a subgroup analysis to assess the association between the type of treatment centre and “not completing treatment,” stratified by types of initial treatment regimen. This was because treatment regimens are a known factor associated with treatment completion (29). We chose 3HR as the reference group, because it was the most prescribed in our population.

## Results

### Overall cascade of care

**FIGURE 2:**
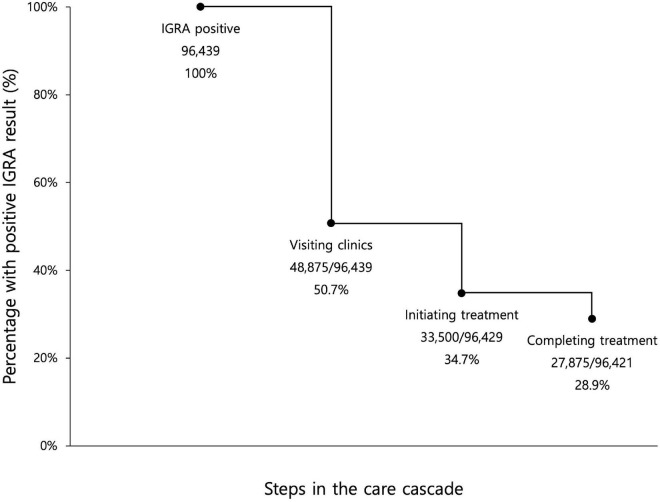
Losses and drop-outs at each stage of the latent tuberculosis cascade of care among all enrolled participants with a positive interferon-gamma release assay. IGRA, interferon-gamma release assay. Eligible participants for the first step, visiting clinics, were all participants with positive IGRA results. Eligible participants for the second step, initiating treatment, were participants with positive IGRA result, who did not have concurrent active TB. Eligible participants for the third step, completing treatment, were participants with positive IGRA result, who did not have concurrent active TB and who did not develop active TB during LTBI treatment.

Among the 96,439 participants with a positive IGRA result, 83,185 (86.3%) were female, and 53,772 (55.8%) were less than 50 years old (Table 1). The percentage subsequently visiting clinics for initial LTBI management was 50.7% (48,875/96,439) (Figure 2). After excluding 10 patients with incident active TB during initial assessment, 33,500/96,429 (34.7%) of the enrolled participants initiated LTBI treatment. After excluding eight patients who developed active TB after treatment initiation, 27,875/96,421 (28.9%) completed treatment.

**TABLE 1:** Multivariable analysis to determine the factors associated with not visiting clinics after a positive interferon-gamma release assay (IGRA) test.

Variables	Total	Not visiting clinic	Univariable analysis		Multivariable analysis	
				
	*n* (column%)	*n* (row%)	RR (95% CI)	*P*-value	aRR (95% CI)	*P*-value
Participants	96,439 (100.0)	47,564 (49.3)				
**Sex**						
Female	83,185 (86.3)	40,156 (48.3)	1		1	
Male	13,254 (13.7)	7,408 (55.9)	1.16 (1.14–1.18)	<0.001	1.11 (1.09–1.13)	<0.001
**Age (years)**						
<20	5,470 (5.7)	2,912 (53.2)	1.18 (1.14–1.22)	<0.001	1.08 (1.04–1.12)	<0.001
20–34	8,003 (8.3)	3,668 (45.8)	1		1	
35–49	40,299 (41.8)	19,326 (48.0)	1.06 (1.03–1.08)	<0.001	1.05 (1.03–1.08)	<0.001
50–64	38,400 (39.8)	19,059 (49.6)	1.09 (1.07–1.12)	<0.001	1.09 (1.06–1.12)	<0.001
≥65	4,267 (4.4)	2,599 (60.9)	1.34 (1.29–1.39)	<0.001	1.30 (1.26–1.35)	<0.001
**Place of residence^a^**						
Rural area	9,481 (9.8)	4,158 (43.9)	1		1	
Small to medium-sized city	33,302 (34.5)	16,968 (51.0)	1.16 (1.13–1.19)	<0.001	1.18 (1.15–1.21)	<0.001
Metropolitan city	53,623 (55.6)	26,416 (49.3)	1.12 (1.10–1.15)	<0.001	1.14 (1.11–1.17)	<0.001
**Income level^b^**						
Low	43,647 (45.3)	20,956 (48.0)	1		1	
Moderate low	26,454 (27.4)	12,553 (47.5)	0.99 (0.97–1.00)	0.153	0.99 (0.98–1.01)	0.484
Moderate high	14,245 (14.8)	7,310 (51.3)	1.07 (1.05–1.09)	<0.001	1.06 (1.04–1.08)	<0.001
High	11,092 (11.5)	6,160 (55.5)	1.16 (1.14–1.18)	<0.001	1.12 (1.10–1.15)	<0.001
**Charlson comorbidity index**						
Score 0	42,088 (43.6)	20,851 (49.5)	1		1	
Score 1	32,208 (33.4)	15,698 (48.7)	0.98 (0.97–1.00)	0.026	0.98 (0.97–1.00)	0.015
Score 2	14,116 (14.6)	6,947 (49.2)	0.99 (0.98–1.01)	0.554	0.98 (0.96–1.00)	0.079
Score 3 or more	8,027 (8.3)	4,068 (50.7)	1.02 (1.00–1.05)	0.047	0.99 (0.97–1.02)	0.523

Multivariable models adjust for all factors in the table. aRR, adjusted relative risk; CI, confidence interval; RR, relative risk. ^a^33 individuals had missing data. ^b^1001 individuals had missing data.

### Attending clinic after a positive test result

**FIGURE 3:**
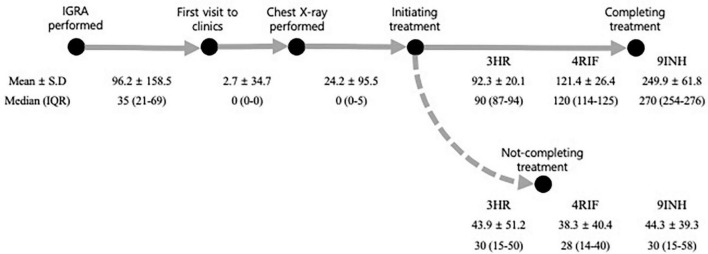
Intervals at each stage of the latent tuberculosis cascade of care. All the numbers were expressed in days. 3HR, 3 months of rifampin and isoniazid combination therapy; IGRA, interferon-gamma release assay; 9INH, 9 months of isoniazid monotherapy; IQR, inter-quartile range; 4RIF, 4 months of rifampin monotherapy; SD, standard deviation.

A median of 35 days and mean of 96.2 days passed between participants performing IGRA and subsequently visiting clinics (Figure 3). In the multivariable analysis, compared to younger age of 20–34 years, all the age groups had a higher likelihood of not visiting clinics, but this effect was particularly prominent in those ≥65 years (adjusted relative risk [aRR], 1.30; 95% confidence interval [CI], 1.26–1.35). Compared to those living in rural area, individuals who lived in small to medium-sized and metropolitan cities were less likely to visit clinics.

### Initiating treatment for latent tuberculosis infection

All the participants who visited clinics had a chest X-ray performed, which occurred after a median of 0 days and a mean of 2.7 days. Among the 48,865 participants who were candidates for LTBI treatment, 33,500 (68.5%) initiated treatment. This occurred within a median of 0 days (IQR of 0–5) and mean of 24.2 days (SD, 95.5) after having a chest X-ray.

**FIGURE 4:**
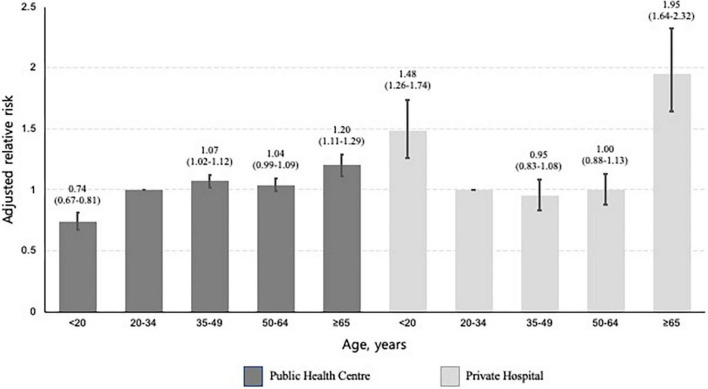
Bar plots presenting the relative risk of not initiating treatment by age, stratified by type of treatment centre. This multivariable analysis was adjusted for sex, place of residence, income level, and Charlson comorbidity index. The baseline category was participants aged 20–34 years. The error bar represents the 95% confidence intervals of each relative risk.

In the multivariable analysis, participants aged ≥ 65 years (aRR, 1.34; 95% CI, 1.25–1.44) were more likely not to initiate treatment, compared to those aged 20–34 years. Compared to private hospitals, public health centres (aRR, 3.72; 95% CI, 3.95–3.86) were associated with treatment non-initiation (Table 2). In a subgroup analysis of those who visited private hospitals, age under 20 was associated with a higher risk of not initiating treatment (aRR, 1.48; 95% CI, 1.26–1.74), compared to being 20–34 years old. In a subgroup analysis of those who visited public health centres, this association was reversed (aRR, 0.74; 95% CI, 0.67–0.81) (Figure 4). In both cases, the oldest age group was associated with an increased likelihood of not initiating treatment.

**TABLE 2:** Multivariable analysis to determine the factors associated with not initiating treatment among participants who visited clinics for initial latent tuberculosis infection management.

Variables	Total	Not initiating treatment	Univariable analysis		Multivariable analysis	
				
	*n* (column%)	*n* (row%)	RR (95% CI)	*P*-value	aRR (95% CI)	*P*-value
Participants	48,865 (100.0)	15,365 (31.4)				
**Sex**						
Female	43,023 (88.0)	13,305 (30.9)	1		1	
Male	5,842 (12.0)	2,060 (35.3)	1.14 (1.10–1.19)	<0.001	1.08 (1.04–1.12)	<0.001
**Age (years)**						
<20	2,556 (5.2)	686 (26.8)	0.88 (0.82–0.96)	0.002	0.91 (0.84–0.99)	0.021
20–34	4,334 (8.9)	1,331 (30.7)	1		1	
35–49	20,971 (42.9)	6,587 (31.4)	1.03 (0.98–1.08)	0.260	1.05 (1.00–1.09)	0.062
50–64	19,336 (39.6)	6,072 (31.4)	1.03 (0.98–1.08)	0.288	1.03 (0.99–1.08)	0.175
≥65	1,668 (3.4)	689 (41.3)	1.35 (1.26–1.46)	<0.001	1.34 (1.25–1.44)	<0.001
**Place of residence^a^**						
Rural area	5,320 (10.9)	1,738 (32.7)	1		1	
Small to medium-sized city	16,332 (33.4)	4,956 (30.3)	0.93 (0.89–0.97)	0.002	1.13 (1.08–1.18)	<0.001
Metropolitan city	27,202 (55.7)	8,665 (31.9)	0.98 (0.93–1.02)	0.240	1.33 (1.28–1.39)	<0.001
**Income level^b^**						
Low	22,688 (46.4)	7,010 (30.9)	1		1	
Moderate low	13,899 (28.4)	4,306 (31.0)	1.00 (0.97–1.04)	0.853	1.01 (0.98–1.04)	0.425
Moderate high	6,932 (14.2)	2,262 (32.6)	1.06 (1.02–1.10)	0.006	1.04 (1.00–1.08)	0.041
High	4,930 (10.1)	1,644 (33.3)	1.08 (1.03–1.13)	0.001	1.08 (1.04–1.13)	<0.001
**Charlson comorbidity index**						
Score 0	21,232 (43.5)	6,762 (31.8)	1		1	
Score 1	16,507 (33.8)	5,096 (30.9)	0.97 (0.94–1.00)	0.044	0.99 (0.96–1.02)	0.456
Score 2	7,168 (1.47)	2,237 (31.2)	0.98 (0.94–1.02)	0.287	1.01 (0.97–1.05)	0.652
Score 3 or more	3,958 (8.1)	1,270 (32.1)	1.00 (0.95–1.05)	0.909	1.04 (0.99–1.09)	0.156
**Type of treatment centre**						
Private hospital	22,733 (46.5)	2,995 (13.2)	1		1	
Public health centre	26,132 (53.5)	12,370 (47.3)	3.61 (3.48–3.74)	<0.001	3.72 (3.59–3.86)	<0.001

Multivariable models adjusted for all factors in the table. aRR, adjusted risk ratio; CI, confidence interval; RR, relative risk. ^a^11 people had missing data. ^b^416 people had missing data.

### Completing latent tuberculosis infection treatment

Among the 33,492 participants who started treatment without developing active TB, the overall percentage completing treatment, not completing treatment, and still being on treatment were 83.2, 14.8, and 2.0%, respectively. The median times between treatment initiation and early termination among 3HR, 4RIF, and 9INH were 30, 28, and 30 days, respectively (Figure 3).

**FIGURE 5:**
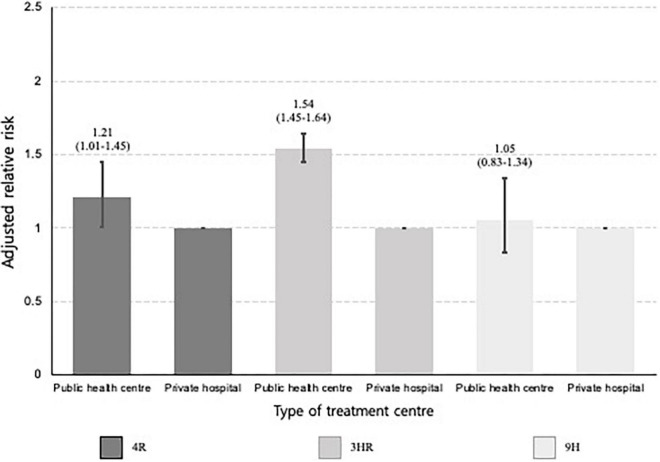
Bar plots presenting the relative risk of not completing treatment by type of treatment centre, stratified by type of initial treatment regimen. 3HR, 3-month of rifampin and isoniazid combination therapy; 9INH, 9-month of isoniazid monotherapy; 4RIF, 4-month of rifampin monotherapy. This multivariable analysis was adjusted for sex, age, place of residence, income level, and the Charlson comorbidity index. The baseline category was private hospital. The error bars represent the 95% confidence interval of each relative risk.

**FIGURE 6:**
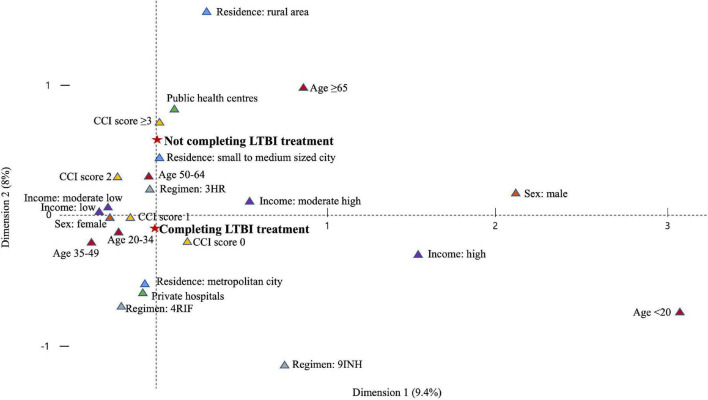
Plot of multiple correspondence analysis representing the relation between not completing treatment and explanatory variables. LTBI, latent tuberculosis infection; CCI, Charlson comorbidity index; 3HR, 3-month of rifampin and isoniazid combination therapy; 9INH, 9-month of isoniazid monotherapy; 4RIF, 4-month of rifampin monotherapy.

In the multivariable analysis, all age categories were associated with a lower risk of not completing treatment versus individuals aged 20–34 years, especially those < 20 years (aRR, 0.52; 95% CI, 0.44–0.61) (Table 3). Individuals who used public health centres (aRR, 1.48; 95% CI, 1.40–1.56) were associated with not completing treatment than those attending private ones. Compared to 3HR, 9H (aRR, 1.28; 95% CI, 1.16–1.41) was associated with a greater likelihood of not completing treatment. Among those who received 3HR, public health centres were associated with not completing treatment (aRR, 1.54; 95% CI, 1.45–1.64) (Figure 5). In the multiple correspondence analysis, we visualised that not completing treatment was associated with attending public health centres, having CCI score ≥ 3, and living in small to medium-sized city (Figure 6). It is negatively associated with 4RIF regimen, attending private hospitals, and living in metropolitan city. However, it is necessary to interpret this multiple correspondence analysis plot with caution because it displays only 17.4% (horizontal axis: 9.4%, vertical axis: 8%) of the variance in the data.

**TABLE 3:** Multivariable analysis to determine the factors associated with not completing latent tuberculosis infection treatment among participants who initiated it.

Variables	Total	Not completing treatment	Univariable analysis		Multivariable analysis	
				
	*n* (column%)	*n* (row%)	RR (95% CI)	*P*-value	aRR (95% CI)	*P*-value
Participants	32,834 (100.0)	4,959 (15.1)				
**Sex**						
Female	29,158 (88.8)	4,377 (15.0)	1		1	
Male	3,676 (11.2)	582 (15.8)	1.06 (0.98–1.15)	0.159	1.10 (1.01–1.20)	0.027
**Age (years)**						
<20	1,816 (5.5)	199 (11.0)	0.58 (0.50–0.67)	<0.001	0.52 (0.44–0.61)	<0.001
20–34	2,938 (8.9)	560 (19.1)	1		1	
35–49	14,092 (42.9)	2,109 (15.0)	0.79 (0.73–0.86)	<0.001	0.79 (0.72–0.86)	<0.001
50–64	13,021 (39.7)	1,955 (15.0)	0.80 (0.73–0.87)	<0.001	0.77 (0.70–0.84)	<0.001
≥65	967 (2.9)	136 (14.1)	0.74 (0.62–0.88)	0.001	0.69 (0.57–0.82)	<0.001
**Place of residence^a^**						
Rural area	3,444 (10.5)	527 (15.3)	1		1	
Small to medium-sized city	11,098 (33.8)	1,893 (17.1)	1.11 (1.02–1.21)	0.021	1.18 (1.08–1.29)	<0.001
Metropolitan city	18,287 (55.7)	2,539 (13.9)	0.91 (0.83–0.99)	0.024	1.05 (0.96–1.15)	0.317
**Income level^b^**						
Low	15,371 (46.8)	2,287 (14.9)	1		1	
Moderate low	9,433 (28.7)	1,389 (14.7)	0.99 (0.93–1.05)	0.744	0.98 (0.92–1.04)	0.528
Moderate high	4,565 (13.9)	758 (16.6)	1.12 (1.04–1.20)	0.004	1.11 (1.03–1.20)	0.007
High	3,202 (9.8)	489 (15.3)	1.03 (0.94–1.12)	0.571	1.08 (0.98–1.18)	0.127
**Charlson comorbidity index**						
Score 0	14,182 (43.2)	2,078 (14.7)	1		1	
Score 1	11,178 (34.0)	1,696 (15.2)	1.04 (0.98–1.11)	0.180	1.04 (0.98–1.11)	0.166
Score 2	4,838 (14.7)	731 (15.1)	1.04 (0.96–1.12)	0.342	1.05 (0.97–1.14)	0.238
Score 3 or more	2,636 (8.0)	454 (17.2)	1.18 (1.08–1.30)	0.001	1.20 (1.09–1.32)	<0.001
**Type of treatment centre**						
Private hospitals	18,992 (57.8)	2,398 (12.6)	1		1	
Public health centres	13,842 (42.2)	2,561 (18.5)	1.47 (1.40–1.55)	<0.001	1.48 (1.40–1.56)	<0.001
**Type of initial regimen**						
3HR	27,118 (82.6)	4,166 (15.4)	1		1	
4RIF	3,555 (10.8)	427 (12.0)	0.78 (0.71–0.85)	<0.001	0.82 (0.75–0.90)	<0.001
9INH	2,161 (6.6)	366 (16.9)	1.10 (1.00–1.22)	0.050	1.28 (1.16–1.41)	<0.001

Multivariable models adjusted for everything in the table. 3HR, 3-months of rifampin and isoniazid combination therapy; 9INH, 9-months of isoniazid monotherapy; 4RIF, 4-months of rifampin monotherapy; aRR, adjusted relative risk; CI, confidence interval; RR, relative risk. ^a^Five people had missing data. ^b^263 people had missing data.

## Discussion

The aim of the current study was to evaluate and monitor the performance of the largest and first LTBI screening project, and which targeted public congregate settings in Korea between 2017 and 2018. We identified where patients were lost to care and the factors associated with these losses. Of individuals diagnosed with LTBI, half attended a clinic for their initial examination and less than one third completed preventive therapy.

The largest drop out arose from people not attending clinics following a positive IGRA test result. It is important to note that participants received their results only via short message service text, and were not given further encouragement or incentives to visit clinics for their initial medical evaluation. As well as this, there are other generally known reasons for non-attendance. These include the low perceived risk of TB infection, and the physical and economic demands of trying to access clinical services (30, 31). For the project to have public health impact, measures need to be introduced that increase participant clinic attendance. For example, in our study older people were less likely to visit clinics than younger people. Older adults rely to a greater degree on general background knowledge and prior experience when making healthcare decisions, whilst younger adults are likelier to engage in an exhaustive review of the available information (32). Thus, each generation is likely to need a different and specific engagement and information strategy.

We considered the type of treatment centre that participants first used as the main exposure of interest for not initiating and not completing treatment. Compared to those who visited private hospitals, people who attended public health centres were less likely to start and complete LTBI treatment. A possible explanation for this may lie with the treating teams in the different sites. For example in public health centres staffing is generally by primary care physicians who might regard treating LTBI as a low priority given the demands of their high general workload (21). However, in private hospitals, most physicians responsible for LTBI management are trained in respiratory medicine, and thus may have greater experience of the importance of its treatment. Therefore, if we wish to optimise care, we need to ensure that there are LTBI education programmes relevant to public health officers in place.

When inclusion of first-grade school students was announced in the current LTBI screening project, there was substantial debate with the Health Education Forum Corporation of South Korea, who objected to this, because first-grade school students were considered to be a low-risk population (33). In our study treatment initiation among participants younger than 20 years, such as first-year high school students, was particularly low, especially if they visited private hospitals. This may be because doctors at private hospitals were reluctant to give preventive therapy to first-grade school students, who were outside the current guideline. However, public health officers were possibly more likely to adhere to the national LTBI strategy – explaining why participants < 20 years who used public health centres, had a higher likelihood of initiating treatment. It is noteworthy that the youngest age group had the highest level of LTBI treatment completion.

As current Korean guideline recommends that people younger than 65 years are tested and treated for LTBI, it is not surprising that we find participants ≥ 65 years being less likely to start treatment. However, if they did take treatment the proportion who successfully completed was similar to that found in other age groups. This suggests that, contrary to long-standing concerns over toxicity, elderly participants were able to take and complete treatment without difficulty. This is in line with other reports (34–36). In a country such as Korea, with its high incidence of active TB among older people, there is a need for preventive therapy to be offered to this population (37). However, before initiating a nationwide roll-out of LTBI preventive therapy in the elderly, more evidence is required to allay fears about safety, and so encourage clinicians to offer treatment.

Within our large, real-world dataset we were able to examine the prescription patterns of LTBI treatment regimens and how commonly patients completed them. The most frequently prescribed regimen was 3HR, followed by 4RIF. The use of RIF-containing preventive therapy is supported by recent studies (38, 39) and guidelines, which reveal higher completion rates and safety levels with comparable efficacy. In addition, we found that, if LTBI treatment were stopped, this most frequently occurred within about 1 month from starting. A possible explanation for this could be the onset of therapy-related adverse events occurring in people who did not feel unwell prior to starting treatment. This early discontinuation of LTBI treatment highlights the importance of sustained adherence support from the outset. In addition, the introduction of safer and shorter regimens will help to further reduce losses during the last steps of the LTBI cascade of care.

In our study, proportion of female participants was high, which reflects female domination of employees at the educational and social welfare institutions in Korea. Interestingly, we observed gender differences of the LTBI cascade of care. Male participants in our study were less likely to visit clinics and start and complete LTBI treatment; however, other studies revealed that female sex was associated with lower rates of treatment initiation in the USA and Canada (36, 40). This could be explained by socio-cultural factors, which affects different health seeking behaviors. For example, women visited their primary care provider to a greater extent than did men for both physical and mental health concerns (41). Another Korean study also revealed that female was prone to initiate treatment among Korean close contacts of active pulmonary TB patients, because they were probably more concerned about having an infection (42). However, gender inequalities in health in other countries might limit women’s access to the healthcare services, which further causes non-adherence to treatment. Further qualitative research is necessary to understand gender differences of the LTBI cascade of care.

We wanted to assess the association between multimorbidity and dropouts, rather than to determine the impact of specific disease on development of TB infection. Our initial hypothesis was that participants with multimorbidity are less likely to start and complete LTBI treatment, because of additional burden of polypharmacy and high likelihood of adverse drug reactions. Our results revealed that comorbidity did not affect initiation of LTBI treatment; however, those with the CCI score of 3 or more were less likely to complete its treatment. This finding suggests that doctors need more caution when providing LTBI treatment to individuals with multiple chronic diseases, such as close and meticulous follow-up through laboratory testing and patient education.

The LTBI cascade of care varies across the globe and depends on geographic settings and target populations. Among close contacts of culture-confirmed pulmonary TB in Brazil between 2015 and 2019, low socioeconomic status and HIV infection were significant determinant of losses in the LTBI cascade of care (43). However, the recent meta-analysis revealed that the cumulative proportion of people living with human immunodeficiency virus (PLHIV) completing TB preventive therapy was higher than previously reported among other at-risk populations (44). Among PLHIV in low- and middle-income countries, overall treatment initiation and completion was similar, regardless of types of LTBI testing. However, among the refugees in the Oregon state of the USA between 2009 and 2012, testing with IGRA had led to significantly higher initiating treatment, compared with tuberculin skin testing (45). Another meta-analysis revealed that initiation and completion of LTBI treatment among the migrants between 2010 and 2020 were higher than before 2010, highlighting improvement of LTBI programs during the last decade (46). These variations suggest diverse and unique barriers and facilitators related to participants demographics and health systems, and it is essential to understand these multifactorial issues to minimise losses along the cascade of care.

This cohort study has several strengths. First, the large sample size provided adequate power for the detection of clinically meaningful factors associated with each step of the LTBI cascade of care. Second, we minimised loss to follow-up by linking together several electronic databases. Third, levels of missing information were also low due to this data linkage.

Despite these strengths, we were limited by the clinical, social and demographic information available to us. For example, details on adverse drug reactions during preventive therapy, which are important reasons for treatment cessation, were not collected. Second, the completion of LTBI treatment was defined by the number of prescriptions of anti-TB drugs, based on insurance claims. Third, various social determinants of health could affect cascade of care, which could limit generalisability of our study. Fourth, it is important to identify specific causes of losses across the LTBI cascade of care, which would guide to prepare public health interventions to minimise drop-out. Because their reasons might vary by regions, time, participants, and cultural backgrounds, it is necessary to conduct additional qualitative study to understand local contexts. However, a qualitative survey was not simultaneously conducted during the nationwide LTBI project to identify them.

As the long-term follow-up of the currently established cohort is possible (19), the priorities of future epidemiological research studies should be: (1) the evaluation of the impact of the programme on the rates of active TB after 2 years among the participants, (2) the assessment of efficacy of three different LTBI regimens preventing development of active TB, (3) the identification of the risk factors associated with active TB progression, such as comorbidities and social determinants of health, and (4) a cost-effectiveness analysis of LTBI screening and treatment within this project.

## Conclusion

The need to expand testing for, and the treatment of, LTBI is critical to meet the WHO End TB Strategy targets. In 2017, the Korean government implemented a nationwide LTBI project targetting congregate settings used by the general public (47). Our study showed that percentage of participants with LTBI who had visited clinics and completed treatment was lower than anticipated. First-year high-school students with a positive IGRA result were less likely to visit clinics for further management, possibly due to controversies within Korea about testing this population. However, completion of LTBI treatment was common among first-year high-school students and elderly participants once they started treatment. Using a public health centre was an important determinant of loss from the LTBI cascade of care, particularly when starting treatment. Regimen completion was most common for rifampin-based, and shorter (3-4 month), treatment regimens. Our research provides important insights for countries using or establishing LTBI programmatic management, as well for those concerned with reducing losses from the LTBI cascade of care, and planning to scale-up treatment for LTBI.

## Data availability statement

The raw data supporting the conclusions of this article will be made available by the authors, without undue reservation.

## Ethics statement

The studies involving human participants were reviewed and approved by Institutional Review Board of Incheon St. Mary’s Hospital, the Catholic University of Korea. Written informed consent from the participants’ legal guardian/next of kin was not required to participate in this study in accordance with the national legislation and the institutional requirements.

## Author contributions

JM, HK, J-PM, HJ, SB, JKa, HY, and JKi: study design. JKi: funding acquisition. HK, YL, J-PM, AS, and JKi: data acquisition. JM, HK, YL, J-PM, HJ, SB, HY, and JKi: data analysis. JM, HK, HS, MR, ML, IA, JKa, S-SL, JP, HY, and JKi: manuscript drafting. JM, HK, HS, MR, ML, IA, HY, and JKi: manuscript revision. All authors read and approved the final manuscript.

## Abbreviations

aRR: adjusted relative risk
IGRA: interferon-gamma release assay
INH: isoniazid
IQR: interquartile range
KCD: Korean classification of disease
LTBI: latent tuberculosis infection
RIF: rifampin
SD: standard deviation
TB: tuberculosis
WHO: World Health Organisation
3HR: isoniazid and rifampin combination therapy for 3 months
4RIF: rifampin monotherapy for 4 months
9INH: isoniazid monotherapy for 9 months.
