# Supplementary material for: The cascade of care for latent tuberculosis infection in congregate settings: A national cohort analysis, Korea, 2017–2018
Source: Front Med (Lausanne). 2022 Sep 15;9:927579. doi: 10.3389/fmed.2022.927579 (PMC9519985; doi:10.3389/fmed.2022.927579)
Supplement: Supplementary file 1 [file Table_1.DOCX]

**S1 Table.** Implications of independent variables for each outcome and their data sources

| **Independent variables** | **Specific outcomes of interest** | | | **Database source** |
| --- | --- | --- | --- | --- |
|  | **Not visiting clinics** | **Not initiating treatment** | **Not completing treatment** |  |
| Sex and Age | • Differences in health perception and health care seeking behaviour | | | • LTBI Screening Database |
| Place of residence | • Limited healthcare resources in rural areas | • Limited healthcare resources in rural areas | • Limited healthcare resources in rural areas | • National Health Information Database |
| Income level | • Socio-economic barriers to access healthcare services | • Lack of awareness of LTBI treatment  • Socio-economic barriers to access healthcare services | • Lack of awareness of LTBI treatment  • Socio-economic barriers to access healthcare services | • National Health Information Database |
| Multimorbidity | • Lots of concerns about illness  • Frequent users of healthcare services | • Additional burden of polypharmacy | • Additional burden of polypharmacy  • High likelihood of side effects | • National Health Information Database |
| Treatment centre visited for initial LTBI management | Not applicable | • Public health officers do not understand importance of LTBI treatment and are not interested in offering this to patients due to their associated increased workloads. | • Public health officers do not know how to manage side effects or non-compliance during LTBI treatment | • LTBI Screening Database |
| Initial LTBI regimens | Not applicable | Not applicable | • Longer treatment duration, more side effects or non-compliance | • National Health Information Database  • Public Healthcare Information System Database |

LTBI, latent tuberculosis infection
